# Biosurfactant from vaginal *Lactobacillus crispatus* BC1 as a promising agent to interfere with *Candida* adhesion

**DOI:** 10.1186/s12934-020-01390-5

**Published:** 2020-06-18

**Authors:** Priscilla Romina De Gregorio, Carola Parolin, Angela Abruzzo, Barbara Luppi, Michele Protti, Laura Mercolini, Jessica Alejandra Silva, Barbara Giordani, Antonella Marangoni, María Elena Fátima Nader-Macías, Beatrice Vitali

**Affiliations:** 1 Centro de Referencia para Lactobacilos (CERELA)-CONICET, Chacabuco, 145, 4000 San Miguel de Tucumán, Tucumán Argentina; 2 Department of Pharmacy and Biotechnologies, University of Bologna, Via San Donato 19/2, 40127 Bologna, Italy; 3 Department of Pharmacy and Biotechnologies, University of Bologna, Via Belmeloro 6, 40126 Bologna, Italy; 4 Microbiology, DIMES, University of Bologna, Via Massarenti 9, 40138 Bologna, Italy

**Keywords:** Vagina, *Lactobacillus crispatus*, *Candida* spp., Biosurfactant, HeLa cells, Murine model, Vulvovaginal candidiasis, Anti-*Candida* activity

## Abstract

**Background:**

*Lactobacillus* spp. dominating the vaginal microbiota of healthy women contribute to the prevention of urogenital and sexually transmitted infections. Their protective role in the vagina can be mediated by *Lactobacillus* cells themselves, metabolites or bacterial components, able to interfere with pathogen adhesion and infectivity. Vulvovaginal candidiasis (VVC) is a common genital infection, caused by the overgrowth of opportunistic *Candida* spp. including *C. albicans, C. glabrata, C. krusei* and *C. tropicalis*. Azole antifungal drugs are not always efficient in resolving VVC and preventing recurrent infections, thus alternative anti-*Candida* agents based on vaginal probiotics have gained more importance. The present work aims to chemically characterize the biosurfactant (BS) isolated from a vaginal *Lactobacillus crispatus* strain, *L. crispatus* BC1, and to investigate its safety and antiadhesive/antimicrobial activity against *Candida* spp., employing in vitro and in vivo assays.

**Results:**

BS isolated from vaginal *L. crispatus* BC1 was characterised as non-homogeneous lipopeptide molecules with a critical micellar concentration value of 2 mg/mL, and good emulsification and mucoadhesive properties. At 1.25 mg/mL, the BS was not cytotoxic and reduced *Candida* strains’ ability to adhere to human cervical epithelial cells, mainly by exclusion mechanism. Moreover, intravaginal (i.va.) inoculation of BS in a murine experimental model was safe and did not perturb vaginal cytology, histology and cultivable vaginal microbiota. In the case of i.va. challenge of mice with *C. albicans*, BS was able to reduce leukocyte influx.

**Conclusions:**

These results indicate that BS from vaginal *L. crispatus* BC1 is able to interfere with *Candida* adhesion in vitro and in vivo, and suggest its potential as a preventive agent to reduce mucosal damage occasioned by *Candida* during VVC.

## Background

*Candida* species are part of the mucosal microbiota of the gastrointestinal and genitourinary tracts of most healthy women. In certain conditions, these yeasts can become opportunistic pathogens and overgrow on the vulvovaginal mucosa, originating symptomatic vulvovaginal candidiasis (VVC). Until some years ago, the species most commonly detected in VVC was *C. albicans*. However, during the last two decades, with the development of more accurate molecular diagnostics, infections by different species (i.e. *C. glabrata, C. krusei* and *C. tropicalis*) have emerged producing an increase in causative species of VVC [1]. Antifungal azoles are usually applied for VVC treatment; however, the prolonged exposition to these drugs can generate high resistance pressure, thus promoting the appearance of azole-resistant *Candida* strains. Thus, the search for anti-*Candida* agents with new pharmacological targets is a priority for effective prevention and treatment of these types of infections [2].

Lactobacilli are the predominant microorganisms in the vaginal microbiota of healthy women that contribute to preventing urogenital and sexually transmitted infections [3–5]. The protective role in the vagina is exerted through different mechanisms including: adhesion and permanence on the epithelium (promoted by biofilm formation), competitive exclusion against pathogens, production of antimicrobial compounds and/or modulation of the host’s immune response [3, 6–9]. In this scenario, probiotics or pharmaceuticals containing lactobacilli or their derivatives represent a novel strategy to reconstitute the vaginal microbiota and prevent/treat urogenital infections [10, 11]. The World Health Organization defines probiotics as “live microorganisms which, when administered in adequate amounts, confer a health benefit on the host” [12, 13]. Pharmabiotics include “live or dead microorganisms, as well as microbial components and metabolites that can interact beneficially with the host” [14].

Different scientists have shown that specific cell surface components of lactobacilli and some metabolites contribute to their beneficial effects [15–19]. Biosurfactants (BS) are amphiphilic compounds produced by microorganisms, anchored on the surface or secreted to the outside, with outstanding surface and emulsifying properties [20]. These molecules, mainly secondary metabolites, exert critical functions in the survival of producer microorganisms by facilitating the transport of nutrients, interfering with microorganism–host interactions and “quorum sensing”, or acting as antimicrobial, anti-adhesive and anti-biofilms agents [15, 16, 21, 22]. Differently from synthetic surfactants, BS are considered “green” compounds because of their natural origin, biodegradability and low toxicity [23], which support their use in medicine. Among pharmaceutical applications, BS antibacterial, antiviral, antifungal and anticancer effects have been reported [21, 24, 25]. Recently, two studies have been focused on the formulation of BS, isolated from a vaginal *L. gasseri* strain, inside liposomes active against *Candida* and methicillin resistant *Staphylococcus aureus* strains [26, 27].

In the perspective to contribute to the understanding of the biological role of lactobacilli in the vaginal niche as well as to promote the application of their isolated components, the present work aims to chemically characterize the BS isolated from a vaginal *Lactobacillus crispatus* strain, and to investigate its cytotoxicity and antiadhesive activity against *Candida* spp., employing in vitro and in vivo assays.

## Methods

### Microorganisms and culture conditions

*Lactobacillus crispatus* BC1, originally isolated from the vaginal swab of a healthy premenopausal volunteer following the protocol approved by the Ethics Committee of the University of Bologna (52/2014/U/Tess) and selected for its antimicrobial properties [28], was used in this study. The strain was cultured in de Man, Rogosa and Sharpe (MRS) (Beckton, Dickinson and Co., Milan, Italy) broth supplemented with 0.05% l-cysteine (Sigma-Aldrich, Milan, Italy), at 37 °C for 24 h in anaerobic jars containing GasPak EZ (Beckton, Dickinson and Co.).

Six clinical isolates of *Candida* spp. (*C. albicans* 1, *C. albicans* 2, *C. albicans* 4, *C. tropicalis*, *C. krusei* and *C. glabrata*), belonging to a collection of yeasts isolated from vaginal swabs of premenopausal, VVC affected-women during routine diagnostic procedures at the Microbiology Laboratory of Sant’Orsola-Malpighi University Hospital of Bologna, were used in the present study [28]. *Candida* strains were grown aerobically at 35 °C for 16 h in Sabouraud dextrose agar (Beckton, Dickinson and Co.).

### Isolation of BS from *L. crispatus* BC1

The cell-bound BS was isolated according to the method previously published by Abruzzo et al. [26]. Briefly, 100 mL of an overnight culture of *L. crispatus* BC1 were inoculated in 900 mL of MRS broth and allowed to grow for 24 h in anaerobic jars containing GasPak EZ. Then, the cell pellet was harvested by centrifugation (10.000×*g*, 10 min), washed twice in sterile water, before being re-suspended in 300 mL of sterile PBS (2.38 g/L Na_2_HPO_4_, 0.19 g/L KH_2_PO_4_ and 8 g/L NaCl) and gently stirred at room temperature for 2 h to release the cell-bound BS. Subsequently the suspension was centrifuged and the supernatant filtered through a 0.22 μm pore size filter (PES 0.22 μm syringe filters, VWR International, Milan, Italy). Cell-free supernatant was then subjected to dialysis against demineralized water in a Cellu-Sep© membrane (molecular weight cut-off 6000–8000 Da; Spectra/Por 2 dialysis membrane, Spectrum Laboratories Inc., Rancho Dominguez, CA) for 24 h at room temperature, and freeze-dried at 0.01 atm and − 45 °C (Christ Freeze Dryer ALPHA 1–2, Milan, Italy). This procedure allowed to obtain about 60 mg of lyophilized BS crude multi-component product (defined as BS) from 1 L of *L. crispatus* BC1 culture.

### Chemical characterization of BS

#### Fourier transformed infrared spectroscopy (FT-IR)

Fourier transformed infrared spectroscopy analyses were conducted with a Jasco FT-IR 4100 spectrophotometer (Jasco, Lecco, Italy) in order to determine the functional groups of the isolated BS. Freeze-dried BS was gently triturated with KBr powder (Sigma-Aldrich) with BS/KBr weight ratio 1:10 and then pressed using a hydraulic press at a pressure of 100 tons for 5 min. The disc was placed in the sample holder and scanned between 4000 and 450 cm^−1^.

#### Mass spectrometric analysis

BS characterisation by mass spectrometry (MS) assays was carried out according to the previously published method by Abruzzo et al. [26], by means of a Waters (Milford, MA, USA) Micromass Quattro Micro triple quadrupole (QqQ) mass spectrometer interfaced with an electrospray ion source operating in positive and negative ionisation modes (ESI+/ESI−). BS was dissolved in ultrapure water and diluted with the same solvent to reach the concentration of 10 μg/mL. BS solutions were directly infused by means of a Harvard Apparatus (Holliston, MA) 11 Plus programmable syringe pump into the ESI source, at a flowrate of 10 μL/min. Data processing was performed using Waters MassLynx 4.1 software. Full scan MS spectra (scan duration: 500 ms) were acquired by applying MS parameters as follows: capillary voltage was set at 3.0 kV, while cone voltage was tested within a range of 15–100 V in order to test variation in BS ionisability and possible fragmentation patterns; source and desolvation temperatures were kept at 120 °C and 150 °C, respectively; cone gas (N_2_) flow was set to 50 L/h while desolvation gas (N_2_) flow was 200 L/h.

### Surface-activity determination and critical micelle concentration of BS

The critical micellar concentration (CMC) of BS was measured by the ring method using a tensiometer (K8600E Krüss GmbH, Hamburg, Germany) equipped with a 1.9 cm platinum ring [26]. Surface tension (dyne/cm) of BS aqueous solutions at different concentrations (0.03–8.0 mg/mL) was measured at room temperature. The concentration at which surfactant molecules aggregate and form micelles in aqueous environment (CMC) was determined by plotting the surface tension as a function of the logarithm of BS concentration. CMC is represented as the point at which the baseline of minimal surface tension intersects the slope where surface tension shows a linear decline.

To evaluate BS stability in a pH range representative of the human vaginal environment in physiological and pathological conditions [4], BS was dissolved (1.25 mg/mL) in aqueous solutions adjusted to pH 3, 4, 5 and 6 with 0.1 M HCl, and the surface tension was determined as described above.

### Emulsification properties of BS

For this study, 1 mL of BS solution (1.25 mg/mL), 4 mL of water and 6 mL of olive oil, sunflower oil or wheat germ oil (Polichimica, Bologna, Italy) were vigorously shaken in graduated glass test tubes for 2 min to obtain maximum emulsification [29]. After 24 h, the height of the emulsion was measured and emulsification index (EI24) was calculated using the following equation:

$$ {\text{EI24}}\left( \% \right) = {\text{Height of emulsion layer}} \times 100/{\text{Total height}} . $$

Water and Tween 80 (Fluka, Milan, Italy) at a concentration of 1 mg/mL were used as negative and positive controls, respectively.

### Mucoadhesive properties

Mucoadhesive properties were determined through turbidimetric analysis of a suspension containing mucin and BS [30]. Mucin (Type II: crude, from porcine stomach, Sigma-Aldrich) was dispersed in water (0.08% w/v) and after 24 h the dispersion was centrifuged at 7500 rpm (GS-15R Centrifuge, Beckman Coulter, Milan, Italy) for 20 min in order to discharge the excess of mucin. Subsequently, the isolated mucin supernatant and a BS solution (1.25 mg/mL) were mixed (1:3 v/v) for 3 h. The turbidity of the mixture was measured at 650 nm (UV–Visible Spectrophotometer, Shimadzu Corporation, Australia) and compared to the controls (mucin supernatant and BS solution without mucin).

### Cytotoxicity of BS on human cervical cells (HeLa)

The impact of BS on the proliferation of HeLa cells was evaluated by colorimetric assay with 3-(45-dimethylthiazol-2-yl)-2,5-diphenyltetrazol (MTT) [31]. HeLa cells were maintained in Dulbecco’s minimal essential medium (DMEM, Sigma-Aldrich), supplemented with 10% fetal bovine serum (Sigma-Aldrich) and 1% l-glutamine (Sigma-Aldrich). Cells were seeded at 10.000 cells/well in 96-well plates and grown to 70% confluence in 5% CO_2_ at 37 °C, in a NU4500E Water-Jacketed CO_2_ Incubator (Nuaire, Plymouth, MN). Subsequently, HeLa cells were treated with different concentrations of BS (0.3–10 mg/mL) and incubated at 37 °C with 5% CO_2_ for 24 h. Untreated cells were used as control. After incubation, 110 μL MTT solution in DMEM (final concentration 0.5 mg/mL) was added to each well and the plates incubated for 4 h at 37 °C with 5% CO_2_. The formazan crystals formed were dissolved by the addition of isopropanol (Sigma-Aldrich). After 15 min, the coloured formazan derivative was quantified by optical density (OD) at 570 nm and the inhibition percent calculated as follows: cell viability (%) = [1 − (OD_sample_/OD_control_)] × 100.

### Anti-adhesive activity of BS against *Candida* spp. in HeLa cells

The influence of BS on the adhesion of *C. albicans* (strains 1, 2 and 4), *C. tropicalis*, *C. krusei* or *C. glabrata* to HeLa cells was determined according to Parolin et al. [28]. Briefly, HeLa cells were grown on sterile glass coverslips placed in 6-well culture plates, up to 70% confluence. Three mechanisms of action: competition, exclusion and displacement, were investigated. For the competition test, 1 mL of *Candida* suspension [1 × 10^8^ colony forming units (CFU) *C. albicans*, *C. krusei* or *C. glabrata*; and 1 × 10^7^ CFU *C. tropicalis*] plus different concentrations of BS (0.00125–1.25 mg/mL) were added to each well and incubated at 37 °C for 1 h. For the exclusion test, the cell monolayers were firstly incubated with 1 mL of BS solutions (0.00125–1.25 mg/mL) for 1 h, and then added with the *Candida* suspensions for 1 h. For the displacement test, the monolayers were incubated first with *Candida* suspensions for 1 h, and then with BS for an additional hour. Once the incubation was finished, the cells were washed with PBS, fixed with May–Grünwald and stained with Giemsa (Sigma-Aldrich). The results were determined by optical microscopy (1000×), quantifying the number of *Candida* bound to HeLa cell. Each adhesion test was performed in duplicate, in two independent experiments, evaluating the fungal number in 200 randomly chosen epithelial cells.

### Murine experimental model

#### Animals

Female BALB/c mice of 2-month-old from the inbred colony of CERELA (Centro de Referencia para Lactobacilos) were employed. Animals were housed and fed ad libitum, and a pseudo-estrous condition was weekly induced as previously described [32]. The experiments were independently repeated two times, with at least five animals for each experimental group and sampling day. The Institutional Laboratory Animal Care and Use Committee of CERELA approved the experimental CRL-BIOT-FB-2019/1A protocol applied in this work.

#### Safety studies

**Fig. 1:**
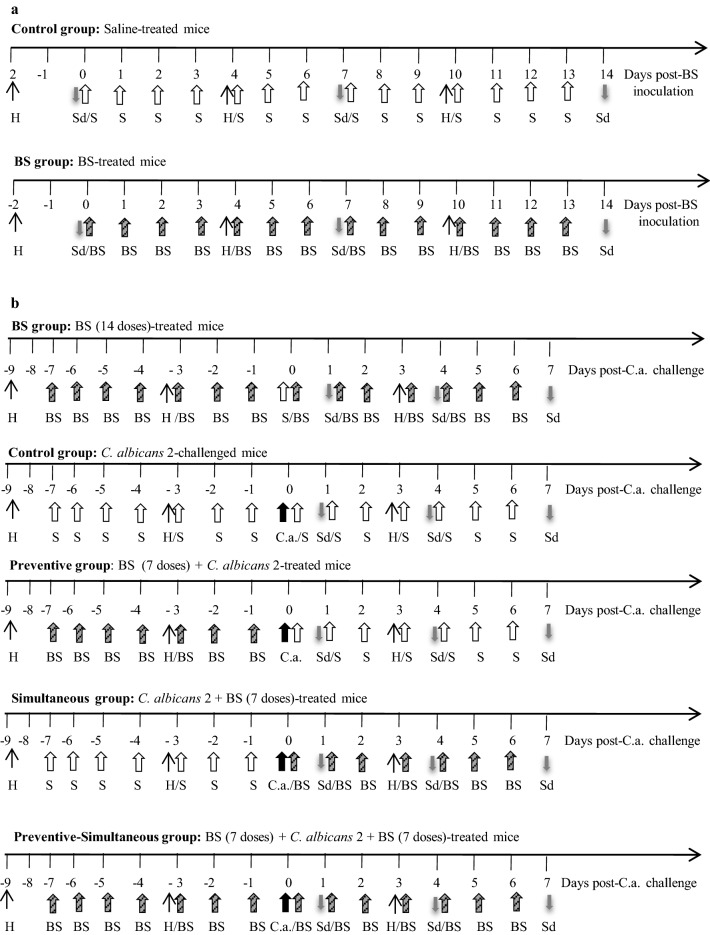
Murine experimental models. **a** Safety assessment of biosurfactant (BS) from *L. crispatus* BC1 in the murine vaginal tract. **b** Preventive, simultaneous and preventive–simultaneous effect of BS against *C. albicans* 2 infection. H: Subcutaneous injection of 0.02 mg β-Estradiol 17-valerate (↑). Arrow correspond to one intravaginal (i.va.) inoculation of: saline (S,  ), BS [20 µL containing 1.25 mg/mL of BS ( )], or *C. albicans* 2 [20 µL containing 10^6^ CFU (C.a.,  )] into BALB/c mice. Sd ( ): sampling day

Mice were randomly assigned to two experimental groups: (a) Control mice: intravaginally (i.va.) inoculated with 20 µL saline once per day for 14 days; (b) BS-inoculated mice: i.va. inoculated with 20 µL BS (1.25 mg/mL in saline) once per day for 14 days. The experimental groups, the inoculation sequence and the sampling days are showed in Fig. 1a.

#### Anti-Candida activity of BS

*Candida albicans* 2 strain was employed in the following experiments to assess anti-*Candida* activity of BS, and the fungal inoculum was prepared as previously described [33].

Mice were randomly assigned to five experimental groups: (a) BS, BS-treated mice: i.va. inoculated with 20 µL BS (1.25 mg/mL) once per day for 7 days, then i.va. inoculated with saline, and again i.va. inoculated with BS once per day for 7 days; (b) Control, *Candida*-challenged mice: i.va. inoculated with saline once per day for 7 days, then i.va. challenged with 20 µL containing 1 × 10^6^ CFU *Candida*, and again i.va. inoculated with saline once per day for 7 days; (c) Preventive, BS + *Candida*-treated mice: i.va. inoculated with 20 µL BS (1.25 mg/mL) once per day for 7 days, then i.va. challenged with 1 × 10^6^ CFU *Candida*, and i.va. inoculated with saline once per day for 7 days; (d) Simultaneous, *Candida* + BS-treated mice: i.va. inoculated with saline once per day for 7 days, then i.va. challenged with 1 × 10^6^ CFU *Candida*, and i.va. inoculated with 20 µL BS (1.25 mg/mL) once per day for 7 days; and (e) Preventive–simultaneous, BS + *Candida* + BS-treated mice: i.va. inoculated with 20 µL BS (1.25 mg/mL) once per day for 7 days, then i.va. challenged with 1 × 10^6^ CFU *Candida*, and again i.va. inoculated with BS once per day for 7 days.

The experimental groups, the inoculation sequence and the sampling days of the protocols applied are schematized in Fig. 1b.

#### Sampling and analytical procedures

Vaginal washings and tissues were obtained according to De Gregorio et al. [32]. Cytology of vaginal washing and histology of vaginal tissues were performed with May–Grünwald–Giemsa and Hematoxylin–Eosin stains, respectively, as previously described [32].

The cultivable bacterial numbers in vaginal washing were determined (only in mice from safety protocol) by the serial dilution method in the following selective agar plates: MRS agar pH 6.4 (Biokar, France), Bile Esculin agar (Britania, Buenos Aires, Argentina), Mannitol Salt Agar (MSA; Britania) and Mc Conkey agar (Britania), to quantify the viable lactic acid bacteria (LAB), enterococci, staphylococci and enterobacteria, respectively, from the murine vaginal microbiota. Fungi were not evaluated because previous studies showed that bacteria, but not fungi were isolated from the vaginal autochthonous microbiota of BALB/c mice [32–34].

In the different experimental protocols for anti-*Candida* activity the following evaluations were performed: (a) *Candida* viable cell numbers by serial dilution method in Sabouraud glucose agar plates containing 0.05 g/L chloramphenicol (Britania) [33]; and (b) average number of leukocytes in vaginal smears (stained with May–Grünwald–Giemsa) counting on optical microscopy in at least 5 fields at 400× magnification.

### Statistical analysis

Student’s t test was applied to compare emulsification properties of BS and Tween 80. Analysis of variance (ANOVA) using a general linear model was applied to analyse the effect of different BS concentrations on: (a) HeLa cell viability, and (b) adhesion of *Candida* spp. to HeLa cells. For in vivo tests, ANOVA (general linear model) was also used to determine the effects of factors (experimental group and sampling day) on the number of viable *Candida* and the leukocyte number. Graphic evaluation of residual values of the significant ANOVAs (*P* < 0.05) showed a distribution close to normal. Then, a Tukey’s test of multiple comparisons was applied, establishing a *P* < 0.05 value to determine the significant differences between mean values, using Minitab statistical software (version 16).

## Results

### Chemical characterization of BS from *L. crispatus* BC1

#### Fourier transformed infrared spectroscopy

**Fig. 2:**
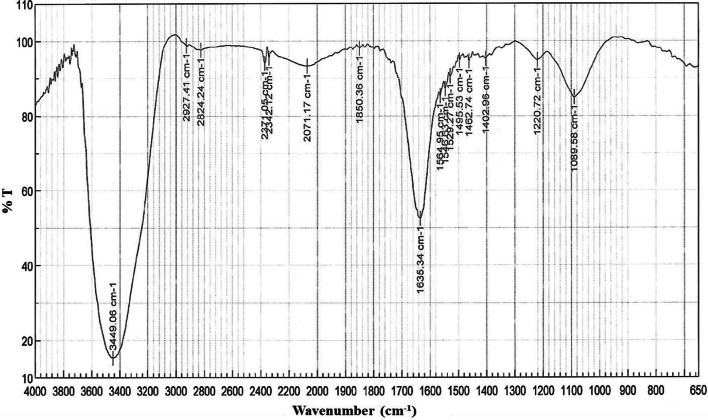
FT-IR spectrum of biosurfactant isolated from *L. crispatus* BC1

BS isolated from *L. crispatus* BC1 was analysed by FT-IR spectroscopy and the obtained spectrum is reported in Fig. 2. The peak at 3449 cm^−1^ was attributed to the stretching of N–H bond [26]. The peaks around 2925 cm^−1^, 1462 cm^−1^ and 2855 cm^−1^ are related to CH_3_ and CH_2_ groups, demonstrating the presence of aliphatic chains [35]. The absorbance at 2300–2400 cm^−1^ may be due to C=N stretching, while the peak at 1635 cm^−1^, relative to CO–N groups, indicated the presence of peptidic groups in the molecule [36]. The peaks at 1564 cm^−1^ and at 1402 cm^−1^ represented the carbonyl group (C=O) of carboxylic acid and C=H stretch [37], respectively. Finally, the absorption band at 1089 cm^−1^ was assigned to O–C–O extend vibrations of carboxylic acids [36].

#### Full scan ESI–MS analysis

BS isolated from *L. crispatus* BC1 strain was analysed by means of a triple quadrupole mass analyser operating in ESI+ and ESI− mode. MS full scan spectra were acquired in an *m/z* range between 20 and 2000 by direct infusion of BS solutions at the concentration of 10 µg/mL in H_2_O, as such and following a mild basic and acid hydrolysis. Pure H_2_O was chosen as solvent for analysis, since the freeze-dried isolated BS powder showed poor solubility in methanol, acetonitrile and mixtures thereof. Acidification of aqueous BS solutions with 0.1% formic acid also gave rise to precipitate formation. MS spectra obtained in negative ionisation mode showed the absence of significant signals within the considered *m/z* range under any of the tested conditions. This suggests the lack of acid functions, typical of glycolipids (mainly rhamnolipids) which usually give rise to signals attributed to negatively charged ions in the 200–700 *m/z* range. Full scan spectra acquired in ESI+ mode, on the other hand, showed significant signals of increasing intensity as the cone voltage was increased (likely due to increased desolvation/ionisation). From the acquired spectra, some predominant signals were highlighted and tentatively attributed to single-charge ions ([M+H]^+^, *m/z* 1397.8), multiple charge ions ([M+2H]^2+^, *m/z* 700.5) and sodium adducts ([M+Na]^+^, *m/z* 1419.6). Within the same *m/z* ranges, minor signals were observed with cluster distribution (spaced by multiples of *m/z* 14) and tentatively attributable to lipid-like portions, thus suggesting to be homologs possessing the same amino acids sequence, but different fatty acid carbon chains. After the hydrolysis of purified BS with HCl in methanol, fatty acid methyl esters were extracted by using ethyl acetate and then analysed. From the obtained full scan MS signals, the putative identity of the main fatty acids has been assigned to β-hydroxytridecanoic acid (3-OH-C13), β-hydroxytetradecanoic acid (3-OH-C14), β-hydroxypentadecanoic acid (3-OH-C15) and β-hydroxyhexadecanoic acid (3-OH-C16). Moreover, both by increasing cone voltage and by analysing BS solutions subjected to mild basic hydrolysis, ion signals could be observed in the 50–200 *m/z* range, compatibly with the progressive fragmentation of amino acid portions attributable to tyrosine, serine, proline, glycine and arginine.

### Surface-activity determination and critical micelle concentration of BS

**Fig. 3:**
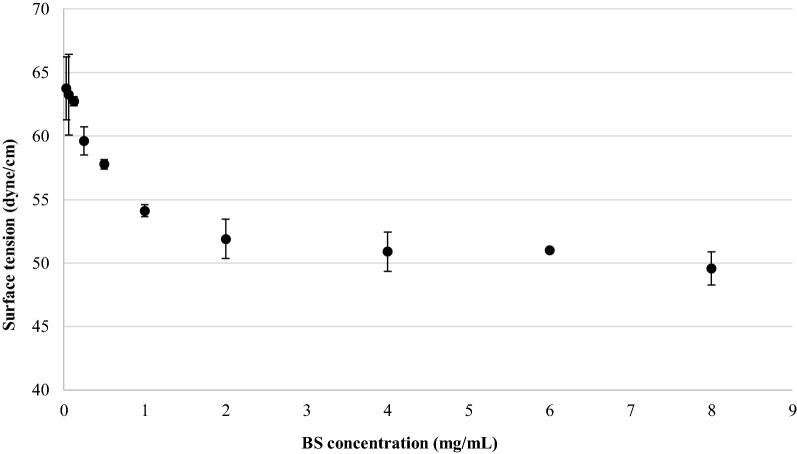
Surface tension versus biosurfactant (BS) concentration (mg/mL). Data are plotted as mean values of surface tension (dyne/cm) ± standard deviation (n = 3)

The surface-active properties of BS mainly depend on its CMC value and its ability to lower surface tension and to stabilize emulsions [38]. BS produced by *L. crispatus* BC1 had the ability to reduce the surface tension from 63.75 ± 2.48 to 49.57 ± 1.31 dyne/cm under the tested concentrations (Fig. 3). Specifically, the surface tension gradually decreased with the increase of BS concentration and no further decrease was observed beyond BS concentration 2.0 mg/mL. The CMC value, calculated by plotting the surface tension as a function of the logarithm of BS concentration, was equal to 2.0 mg/mL. Considering that the human vaginal pH ranges from less than 4 to greater than 5 [4], the surface tension was also measured at pH ranging from 3 to 6. No significant difference (*P* > 0.05) was observed between the surface tension values obtained in water (53.67 ± 0.58 dyne/cm) and in aqueous solutions at pH 3, 4 and 5 (data not shown). At pH 6.0, a slightly lower surface tension (50.67 ± 1.15 dyne/cm) with respect to the values obtained in water and at pH 3 or 4 (*P* < 0.05) was obtained. These results highlighted the maintenance of the surfactant properties over a pH range from 3 to 6 and suggested the effectiveness of BS for vaginal application.

### Emulsification properties of BS

**Fig. 4:**
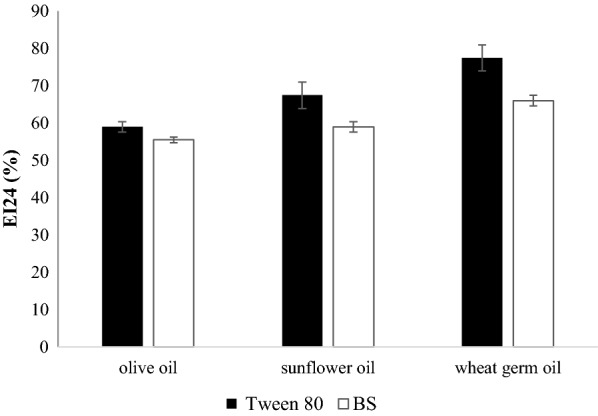
Emulsification properties of biosurfactant (BS) from *L. crispatus* BC1. Emulsification index at 24 h (EI24%) of BS and Tween 80 calculated on different substrates: olive oil, sunflower oil and wheat germ oil. Data are plotted as mean values of EI24% ± standard deviation (n = 3)

The emulsification activity of BS was determined against olive oil, sunflower oil and wheat germ oil. The maximum emulsification activity of BS was 66.0 ± 1.4% for wheat germ oil, followed by sunflower oil and olive oil (Fig. 4). In all cases, EI24 was higher than 50% and only slightly lower (*P *> 0.05) than that obtained with Tween 80 (used as control).

### Mucoadhesive properties of BS

Mucoadhesive properties of BS were investigated by measuring the turbidity at 650 nm of the mixture BS/mucin, compared to the turbidity of BS solution and mucin supernatant. The mixture of BS and mucin was characterized by a higher turbidity with respect to the controls, as a consequence of the interaction between BS and mucin [26]. Specifically, after 3 h the percentage increase of the turbidity was equal to 122 ± 2%.

### Cytotoxic effect of BS on HeLa cells

**Fig. 5:**
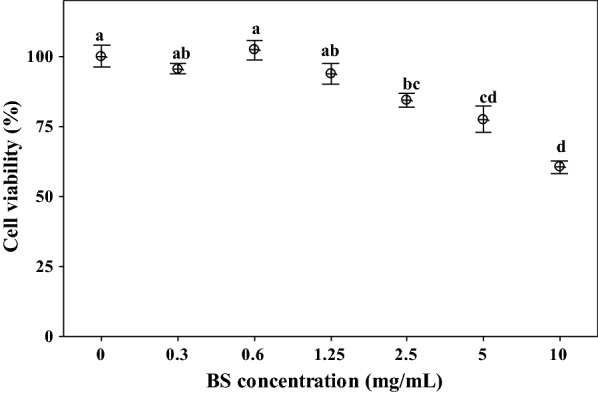
Viability of cervix epithelial cells (HeLa) in presence of biosurfactant (BS) from *L. crispatus* BC1. Data are plotted as average values of cell viability (%) ± standard error. Statistically significant differences in mean values of cell viability (%) are indicated by different letters (*P* < 0.05)

Before studying the potential of BS towards *Candida* adhesion on HeLa cells, we assessed if BS could interfere with cell viability in this cellular model. Thus, HeLa cells were treated with different concentrations of BS for 24 h, and then subjected to MTT reduction assay. Concentrations of BS below 2.5 mg/mL did not significantly reduce cell viability. For higher BS concentrations, a dose-dependent reduction (*P* < 0.01) was observed. In particular, BS at the concentration of 2.5 mg/mL reduced cell viability by 15.63%; 5 mg/mL by 22.52%; and 10 mg/mL decreased viability by 39.43% (Fig. 5).

### Anti-adhesive activity of BS against *Candida* spp

**Fig. 6:**
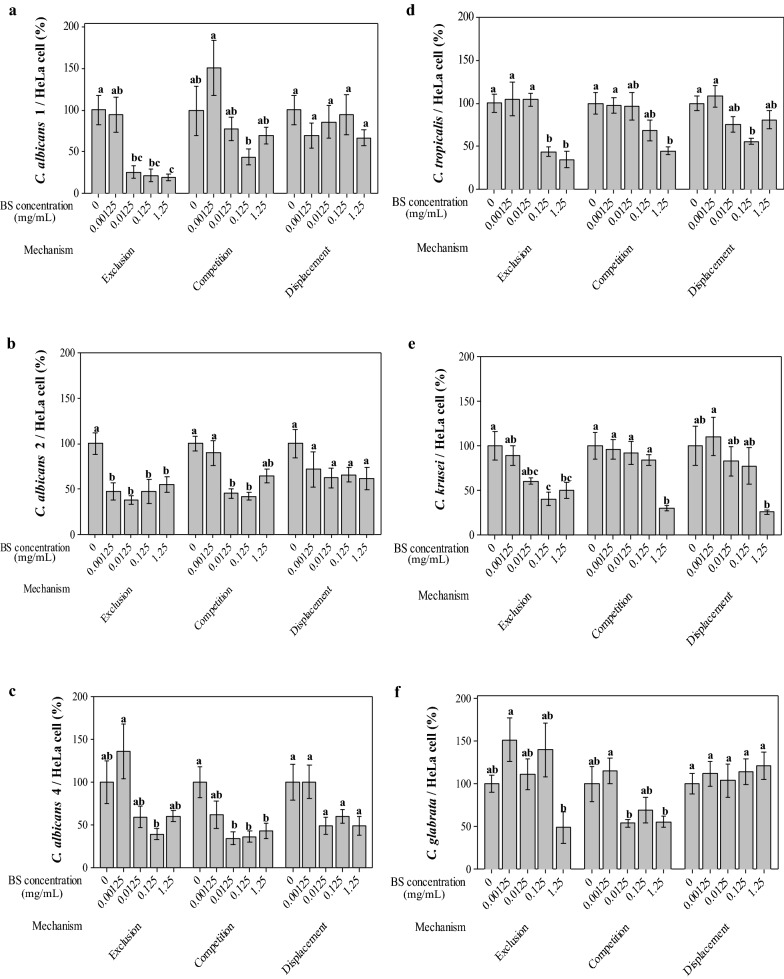
Interference of biosurfactant (BS) from *L. crispatus* BC1 with *Candida* adhesion to HeLa cells. *C. albicans* 1, 2 and 4 (**a**–**c**), *C. tropicalis* (**d**) *C. krusei* (**e**) and *C. glabrata* (**f**). Exclusion, competition and displacement mechanisms were studied. The results were expresses as percentages of adherent yeasts per HeLa cell and compared to adhesion without BS (control value), taken as 100%. Data are plotted as average values of number of *Candida*/HeLa cells (%) ± standard error. Statistically significant differences in mean values in each test (exclusion, competition, displacement) are indicated by different letters (*P* < 0.05)

In order to evaluate the antagonist effect of BS against *Candida*, the influence of different concentrations of BS (0.00125–1.25 mg/mL) on the adhesion of *C. albicans, C. tropicalis, C. krusei* and *C. glabrata* to HeLa cells was investigated (Fig. 6) by using exclusion, competition and displacement assays. The BS concentrations were selected on the basis of the previous experiment, as they did not affect HeLa cell proliferation.

The activity of BS against yeast adhesion to HeLa cells showed to be *Candida* strain-specific because different results were observed among strains of the same species (*C. albicans*) (Fig. 6a–c), and different species (*C. tropicalis*, *C. krusei* and *C. glabrata*) (Fig. 6d–f). At the lowest dose of BS (0.00125 mg/mL), for some *Candida* strains, an apparent not-significant enhancement of *Candida* adhesion was observed, probably attributable to experimental variability or to a slight substrate-like effect of BS. For higher concentrations of BS, an inhibitory activity was demonstrated, being BS more effective by exclusion mechanism than by competition and displacement mechanisms.

In the exclusion test, a significant reduction of at least 50% was evidenced in the adhesion of *C. albicans*, *C. tropicalis* and *C. krusei* strains to HeLa cells at BS concentration of 0.125 mg/mL (Fig. 6a–e). *C. albicans* 1 and 2 adhesion was reduced even with lower concentrations (0.0125 and 0.00125 mg/mL, respectively) (Fig. 6a, b).

When evaluating the competition mechanism, the adhesion of *C. albicans* 2 and 4 to HeLa cells was significantly reduced by at least 50% with 0.0125 and 0.125 mg/mL BS (Fig. 6b, c). This effect was also evidenced for *C. albicans* 1 with 0.125 mg/mL BS and for *C. tropicalis* and *C. krusei* with 1.25 mg/mL BS. For *C. glabrata*, adhesion was decreased with BS concentrations ranging from 0.0125 to 1.25 mg/mL; however, this reduction was not significant compared to control, demonstrating that *C. glabrata* is the most resistant strain/species (Fig. 6f).

In the displacement test, a significant reduction in the adhesion of *C. tropicalis* and *C. krusei* to HeLa cells was only evidenced in the presence of 0.125 and 1.25 mg/mL BS, respectively, compared to their controls. The BS was not able to interfere with the adhesion of the other tested *Candida* strains in the displacement protocols.

### Studies in murine experimental models

The concentration of 1.25 mg/mL of BS was selected to evaluate the anti-*Candida* activity in a murine experimental model, taking into account that this was the most effective dose in reducing *Candida* adhesion to HeLa cells, by the different mechanisms studied.

#### Safety assay of BS

In order to assess the safety of BS, mice were i.va. inoculated for 14 days for cytological and histological evaluation of the murine vaginal tract. The analysis evidenced no adverse effect of BS (Additional file 1: Fig. S1a, b). The May–Grünwald–Giemsa-stained vaginal smears revealed a vaginal discharge of cornified surface cells with pyknotic nucleus or flakes and keratinized cell groups in both control and BS-treated mice (Additional file 1: Fig. S1a). Also, the characteristics of the vaginal epithelium and lamina propria were similar in BS-treated and control groups at days 7 and 14 post-BS inoculation (Additional file 1: Fig. S1b).

#### Effect of BS on cultivable murine vaginal microbiota

The i.va. inoculation of BS did not significantly affect the number of viable lactic acid bacteria, enterococci, staphylococci, and enterobacteria, although a decrease in the number of viable enterobacteria was observed at day 14 post-BS inoculation compared to control mice (*P* > 0.05) (Additional file 1: Fig. S1c).

#### Anti-Candida activity of BS in mice experimental model

The effect of i.va. administration of BS by preventive, simultaneous and preventive–simultaneous protocols against i.va. challenge with *C. albicans* 2 was determined. The *C. albicans* 2 strain was employed in these assays, since it was characterised by the lowest adhesion to HeLa cells in the presence of BS.

**Fig. 7:**
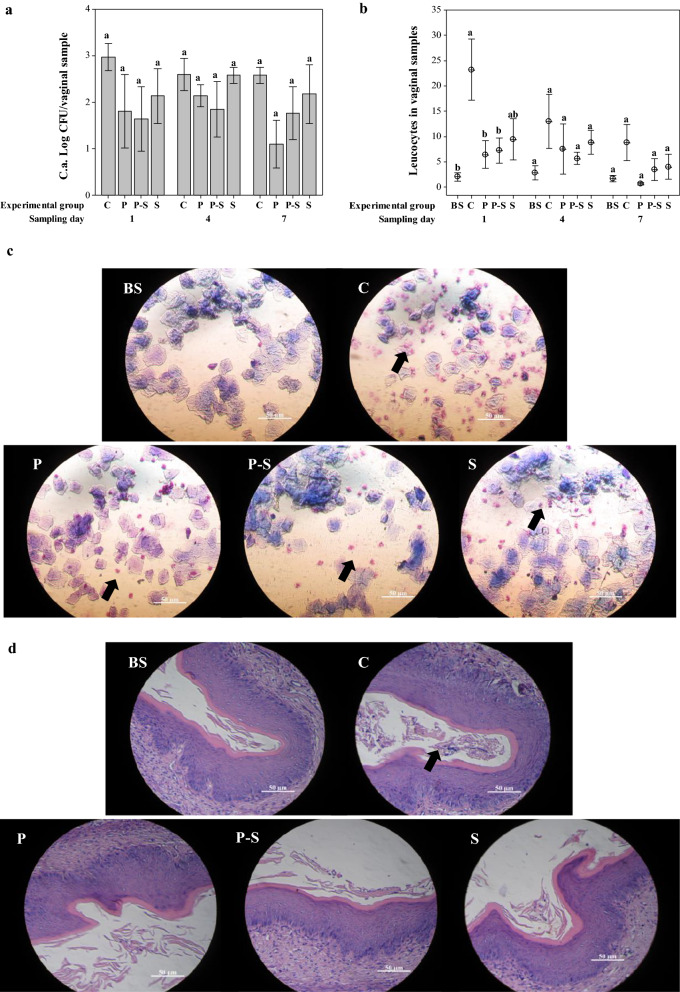
Anti-*Candida* activity of biosurfactant (BS) from *L. crispatus* BC1 in a murine experimental model. **a***C. albicans* 2 (C.a.) viable cells and **b** leukocytes in vaginal samples of mice receiving preventive (P), preventive–simultaneous (P–S) or simultaneous (S) intravaginally (i.va.) administration of BS against i.va. challenge with C.a. C and BS correspond to mice inoculated only with C.a. and BS, respectively. Data are plotted as average values of C.a. viable cell or leukocyte numbers ± standard error. Statistically significant differences between the values of experimental groups on the same sampling day are indicated by different letters (*P* < 0.05). **c** May Grunwald–Giemsa-stained vaginal smears and **d** Hematoxylin–Eosin-stained vaginal slides of the different experimental group at day 1 of sampling. Influx of leukocytes in the vaginal washing and lumen of C.a.-challenged mice is indicated with black arrows. Results are representative of two independent experiments

*Candida* viable cells were enumerated in mice vaginal samples obtained from the different BS protocols against i.va. *C. albicans* 2 challenge, but a significant inhibitory effect was not observed. However, the lowest *C. albicans* 2 viable cell counts were observed in the preventive and preventive-simultaneous protocols at all the sampling days compared to controls (Fig. 7a).

The cytological and histological studies of the murine vaginal tract demonstrated that the challenge with *C. albicans* 2 induced a leukocyte influx at all the sampling days (Fig. 7b–d). Notably, this influx was reduced by BS treatments at all the sampling days, being this reduction significant on day 1 post-C.a. challenge in preventive and preventive-simultaneous protocols (Fig. 7b–d).

## Discussion

The antibacterial, antifungal and antiviral effect of some BS from lactobacilli have previously been reported [8, 15, 25, 39–43]. Also, another interesting application attributed to BS from lactic acid bacteria is their use as anti-adhesive agents to prevent pathogen adhesion to the host epithelium and to solid surfaces as biomedical instruments [42–49]. In this context, some authors showed that the action of BS from lactobacilli against *Candida* is most likely related to anti-adhesive and anti-biofilm effects rather than to antimicrobial activity [43, 45]. However, the anti-adhesive properties of BS are frequently determined in in vitro models on polystyrene plates, although the use of plates does not mimic the in vivo conditions of epithelial cells [39, 42, 43, 45]. Moreover, the safety of BS must be assessed before its application in humans, and in vitro cytotoxicity assays and experimental animal models are available [43]. In the present work, a new BS was isolated from a vaginal *L. crispatus* strain, and its anti-adhesive effect against *Candida* spp. were investigated on human epithelial cells and, for the first time, in a murine experimental model. Also, the chemical characterization of BS was carried out in order to define the structure and/or composition of this biomolecule. The employment of a vaginal-derived BS to prevent or treat *Candida* infection in the vaginal environment represents the main novelty of this study.

The results of FT-IR analysis demonstrated a non-homogeneous chemical composition and a lipopeptidic nature of the BS isolated from *L. crispatus* BC1. Moreover, BS possesses surface tension property along with a CMC value corresponding to 2 mg/mL. This result was in agreement with previous reports of BS produced by lactobacilli [16, 35, 49] or synthetic surfactant, such as sodium dodecyl sulphate (SDS) [50]. In addition, a good emulsification property of BS against olive oil, sunflower oil and wheat germ oil was demonstrated. As regard to the CMC and the emulsification property, similar results were also determined in our previous work, where BS isolated from *L. gasseri* BC9 showed a high surface activity and a CMC value corresponding to 2.5 mg/mL [26].

Even though the biosurfactant was obtained from a Generally Recognized As Safe (GRAS) microorganism, in the present work we evaluated BS cytotoxicity on human epithelial cells and BS turned to be non-cytotoxic at the concentration of 1.25 mg/mL. Higher BS concentrations significantly decreased cell viability, possibly due to a detergent-like effect leading to cell membrane disruption [35, 51, 52]. Similarly, Ferreira et al. [53] evaluated the cytotoxicity of a BS from *L. paracasei* using a mouse fibroblast cell line and demonstrated that 5 and 10 mg/mL BS showed cell proliferation values of 97% and 64%, respectively, whereas 0.5 mg/mL SDS, a synthetic well known surfactant, showed a strong inhibitory effect. Sharma et al. [35] have also studied the cytotoxicity of BS from *L. helveticus* on a mouse fibroblast cell line, but the results demonstrated 43.3% cell viability at 0.0625 mg/mL BS, showing similar toxicity when compared with SDS. On the other hand, Chiewpattanaku et al. [24] evaluated the cytotoxicity of a BS produced by *Exophiala dermatitidis* fungus against tumor cell lines [HeLa and leukemia (U937)] and normal cell lines [African green monkey kidney (Vero) and peripheral blood mononuclear cells (PBMC)]. The authors reported the anti-proliferative activity against HeLa and U937 cell lines [Inhibitory concentration 50 (IC_50_) 0.029 mg/mL and 0.049.85 mg/mL, respectively] in a dose-dependent manner and no cytotoxicity in normal cells even when high concentrations were used. The results obtained in this work indicate that 1.25 mg/mL BS from *L. crispatus* BC1 is not cytotoxic to HeLa cells.

The therapy and control of infections produced by *Candida* species require strategies combining antifungal agents with substances that block the adhesion to host cells or medical device surfaces [54]. Biosurfactants can adsorb to surfaces, forming a film at the interfaces by orienting polar and nonpolar groups according to the surface hydrophilicity/hydrophobicity. The interaction between BS and a substratum surface alters the hydrophobicity, thereby interfering with microbial adhesion and desorption processes [55, 56]. Thus, the application of BS from lactobacilli could disturb *Candida* adhesion and desorption processes by interfering with hydrophobicity [25, 43]. The inhibition potential of BS from *L. crispatus* BC1 on *Candida* adhesion was assayed on cervix epithelial cells (HeLa) since this cell line represents a well-established model to study *Lactobacillus/Candida* adhesion, and, previously, such anti-*Candida* activity was demonstrated for *L. crispatus* BC1 whole cells on the same cellular model [28]. Adhesion assays showed that BS from *L. crispatus* BC1 was able to decrease the adhesion of all the *Candida* strains tested. However, differences in the adhesion were evidenced among strains of the same species (*C. albicans*), and of different species (*C. tropicalis*, *C. krusei* and *C. glabrata*). In this way, BS could induce changes in the cell surface characteristics and modify the interface contributing to the inhibition of the adherence of yeast to the epithelial cells. In addition, the cell surface differences in carbohydrate and protein concentrations between the *Candida* strains could contribute to the modulation of cell adhesion [57].

When evaluating the mechanisms by which the BS from *L. crispatus* BC1 interferes with the adhesion of *Candida* spp. to HeLa cells, the exclusion assay showed to be the most effective. In a similar way, Itapary Dos Santos et al. [43] demonstrated that BS isolated from *Lactobacillus* reference strains (*L. rhamnosus* ATCC 9595 and *L. acidophilus* ATCC 4356) and vaginal (*L. paracasei* 11 and *L. gasseri* 1) reduced *C. albicans* adhesion and disrupted the biofilm formation on polystyrene plates, obtaining better results in pre-incubation assay than in co-incubation experiments. Thus, the results suggest the potential use of BS from *L. crispatus* BC1 as a pharmaceutical ingredient for the prevention of recurrent VVC, because the long-term azole treatment (producing longer asymptomatic periods between recurrences) does not provide a definitive cure [58, 59].

In order to corroborate the results obtained from in vitro studies and to overcome the limits of the employment of cervical cells, a murine experimental model was employed. In a first step, the i.va. inoculation of 7 and 14 doses BS did not produce modification in the murine vaginal cytology and histology and did not significantly affect the number of cultivable bacteria evaluated in vaginal washings. These results suggest that the i.va. application of BS (1.25 mg/mL) is safe and does not modify the cultivable vaginal microbiota. Similar to the present work, analytical methods of cultivation and isolation were widely applied in several laboratory animals in order to evaluate their vaginal microbiota [32, 34, 60–62]. Taking into account that the cultured-based technique used in the present work does not allow the isolation of uncultured microorganisms, further studies should be carried out by applying molecular methodologies to evaluate the effect of BS from *L. crispatus* BC1 on different murine vaginal microbial populations.

As member of the normal human microbiota, *C. albicans* commonly colonizes the vaginal lumen asymptomatically [63]. However, symptomatic infection can result from exuberant mucosal inflammation that is caused primarily by fungal overgrowth in the vagina and subsequent epithelial invasion and production of virulence effectors [64]. The yeast-to-hypha switch under morphogenesis-inducing conditions (e.g., increases in estrogen, elevated vaginal pH, and microbiome disruption) induces an augmented recognition by pattern recognition receptors (PRR) on the epithelial surface, increased hyphal biomass, expression of hypha-associated virulence factors that activates NLRP3 inflammasome signaling, eliciting inflammatory cytokines and chemokines in the vaginal epithelium, resulting in initial migration of polymorphonuclear leukocytes (PMNs) from the lamina propria to the vaginal lumen. The failure to adequately reduce immunopathological triggers results in the continued expression of innate immune effectors by the vaginal epithelium. These initial signals, coupled with the secondary amplification of immune effectors by recruited PMNs, contribute to symptomatic infection and characteristic immunopathology [65]. Peters et al. [66] have demonstrated that the depletion of PMNs with neutralizing antibodies neither reduced fungal load nor mucosal damage levels during infection, suggesting that neutrophils are non-protective under such conditions and that mucosal damage is mediated by the fungus. Thus, it has been suggest that targeting the NLRP3 inflammasome during VVC may be a rational therapeutic option for disease management [64]. When evaluating the preventive, simultaneous and preventive-simultaneous effect of BS against the i.va. challenge with *C. albicans* 2 in the murine experimental model, a significant decrease in *Candida* colonization was not observed with the different protocols applied. However, a significant reduction in the leukocyte influx induced by the fungus was evidenced with the preventive and preventive–simultaneous administration of BS (Fig. 7a, b). The ability of the BS molecule to interact with mucin could imply an increase of the residence time at the application site and, consequently, a longer contact period with the vaginal mucosa [67]. Thus, the decrease of the leukocyte influx induced by *C. albicans* 2 strain when preventive and preventive-simultaneous protocols were applied could be explained by the interaction of BS with the mucin secreted by the vaginal epithelium, that could delay the contact of the fungus with the epithelium. Thus, the results suggest that BS preventive application could avoid the mucosal damage mediated by *C. albicans* during VVC, consequently reducing the high levels of inflammatory cytokines in the genital tract that could cause mucosal vulnerability and increased risk of HIV and sexually transmitted infection (STI) acquisition [68].

In the present work a murine model was employed to evaluate the host–*Candida/*BS interactions in the vaginal environment. We are aware that there are some differences between physiology and characteristic of humans and our murine model counterpart, such as vaginal pH, native microbiota, type and duration of sexual cycle, and immune repertoire [69]. In general, low amounts or absence of lactobacilli and a neutral pH in murine vagina are reported, unlike the high number of lactobacilli and low pH, which are characteristics of the human vagina [70]. However, given the success of other described vaginal disease mouse models [32, 33, 69, 71–73], and our findings thus far, we believe this model has useful applications in studying host–*Candida* or BS interactions in the vaginal environment. To the best of our knowledge, the effect of BS isolated from vaginal lactobacilli on *Candida albicans* in a murine experimental model was not reported to date.

## Conclusions

In this study, a biosurfactant produced by vaginal *L. crispatus* BC1 was chemically and biologically characterized. The BS presented a non-homogeneous chemical composition and a lipopeptide nature, a CMC value corresponding to 2 mg/mL, and a good emulsification capability against olive oil, sunflower oil, and wheat germ oil. Moreover, BS reduced the *Candida* strains’ ability to adhere to human cervical epithelial cells, mainly by exclusion mechanism. Employing a murine experimental model, it was evidenced that the i.va. inoculation of BS did not affect the vaginal cytology, histology and vaginal microbiota (cultivable); but reduced the influx the leukocytes induced by the i.va. challenge with *C. albicans*. This study represents the first characterization of BS from vaginal *L. crispatus* BC1 and its potential to interfere with *Candida* adhesion and the mucosal inflammation mediated by the fungus during VVC. However, further studies are required to deeply investigate such properties of BS and its potential use in humans.

## Supplementary information

**Additional file 1: Fig. S1.** Effect of biosurfactant (BS) from *L. crispatus* BC1 on murine vaginal cytology, histology and microbiota. Photographs of (a) May Grunwald–Giemsa-stained vaginal smears and (b) Hematoxylin–Eosin-stained vaginal slides from BALB/c mice intravaginally (i.va.) inoculated with 20 µL of saline (control mice) or 20 µL of biosurfactant from *L. crispatus* BC1 (BS, 1.25 mg/mL) (BS-treated mice), for 7 or 14 days. Results are representative of two independent experiments. (c) Viable cells of lactic acid bacteria, enterococci, staphylococci and enterobacteria from murine vaginal washings (v.w.) of the two experimental groups (BS and control) at days 0, 7 and 14 post-BS inoculation. The data are plotted as the mean values of viable cell numbers (Log CFU/mL) ± standard error.

## Data Availability

All data generated or analysed during this study are included in this published article and its additional information files.

## Abbreviations

VVC: Vulvovaginal candidiasis
BS: Biosurfactant
i.va.: Intravaginal
MRS: Man, Rogosa and Sharpe
FT-IR: Fourier transformed infrared spectroscopy
MS: Mass spectrometry
CMC: Critical micellar concentration
EI24: Emulsification index
HeLa: Human cervical cells
MTT: 3-(45-dimethylthiazol-2-yl)-2,5-diphenyltetrazol
DMEM: Dulbecco’s minimal essential medium
CFU: Colony forming units
ANOVA: Analysis of variance
SDS: Sodium dodecyl sulphate
C.a.: *Candida albicans*
P: Preventive
P–S: Preventive–simultaneous
S: Simultaneous
PMNs: Polymorphonuclear leukocytes
