# Supplementary figures and images for: Biosurfactant from vaginal Lactobacillus crispatus BC1 as a promising agent to interfere with Candida adhesion
Source: Microb Cell Fact. 2020 Jun 18;19:133. doi: 10.1186/s12934-020-01390-5 (PMC7302142; doi:10.1186/s12934-020-01390-5)

**a**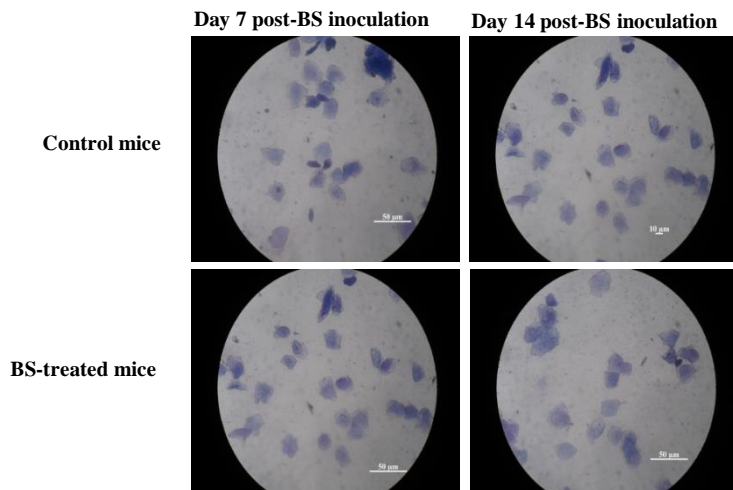**b**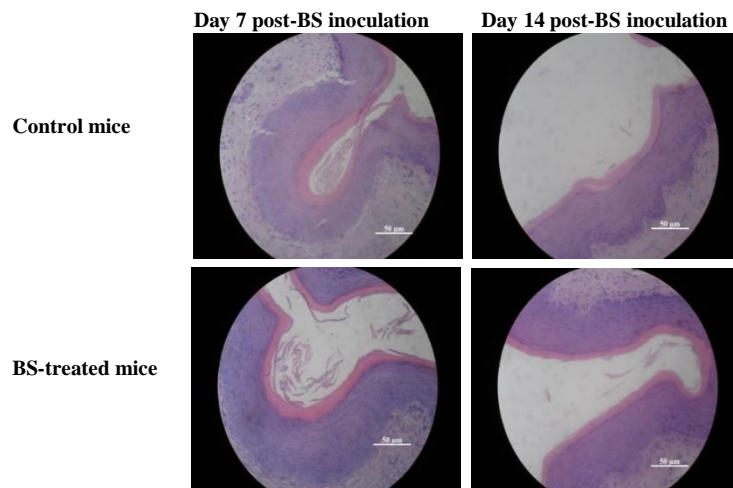**c**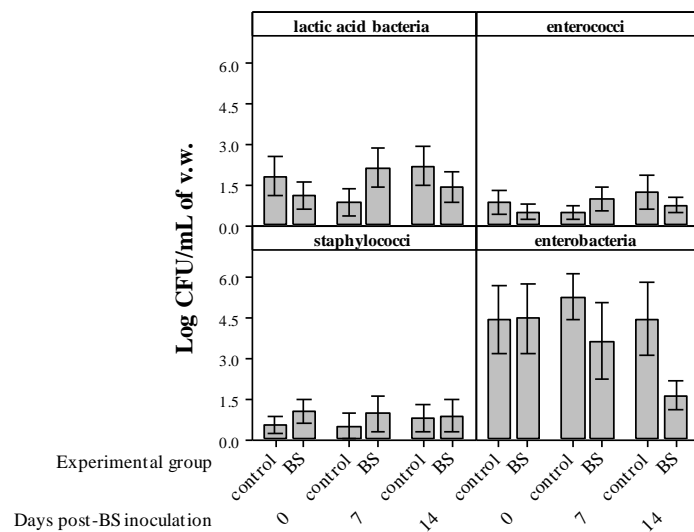

Supplement: Supplementary file 1 — Additional file 1: Fig. S1. Effect of biosurfactant (BS) from L. crispatus BC1 on murine vaginal cytology, histology and microbiota. Photographs of (a) May Grunwald–Giemsa-stained vaginal smears and (b) Hematoxylin–Eosin-stained vaginal slides from BALB/c mice intravaginally (i.va.) inoculated with 20 µL of saline (control mice) or 20 µL of biosurfactant from L. crispatus BC1 (BS, 1.25 mg/mL) (BS-treated mice), for 7 or 14 days. Results are representative of two independent experiments. (c) Viable cells of lactic acid bacteria, enterococci, staphylococci and enterobacteria from murine vaginal washings (v.w.) of the two experimental groups (BS and control) at days 0, 7 and 14 post-BS inoculation. The data are plotted as the mean values of viable cell numbers (Log CFU/mL) ± standard error. [file 12934_2020_1390_MOESM1_ESM.pdf]
